# Fever‐Inspired Immunotherapy Based on Photothermal CpG Nanotherapeutics: The Critical Role of Mild Heat in Regulating Tumor Microenvironment

**DOI:** 10.1002/advs.201700805

**Published:** 2018-03-25

**Authors:** Yan Li, Lianghua He, Haiqing Dong, Yiqiong Liu, Kun Wang, Ang Li, Tianbin Ren, Donglu Shi, Yongyong Li

**Affiliations:** ^1^ Shanghai East Hospital The Institute for Biomedical Engineering and Nano Science Tongji University School of Medicine Shanghai 200092 P. R. China; ^2^ School of Materials Science and Engineering Tongji University 4800 Caoan Road Shanghai 201804 P. R. China; ^3^ School of Life Science and Technology Tongji University 1239 Siping Road Shanghai 200092 P. R. China; ^4^ The Materials Science and Engineering Program Department of Mechanical and Materials Engineering College of Engineering and Applied Science University of Cincinnati Cincinnati OH 45221 USA

**Keywords:** CpG, immunotherapy, mild heat, nanotherapeutics, tumor microenvironments

## Abstract

Although there have been more than 100 clinical trials, CpG‐based immunotherapy has been seriously hindered by complications in the immunosuppressive microenvironment of established tumors. Inspired by the decisive role of fever upon systemic immunity, a photothermal CpG nanotherapeutics (PCN) method with the capability to induce an immunofavorable tumor microenvironment by casting a fever‐relevant heat (43 °C) in the tumor region is developed. High‐throughput gene profile analysis identifies nine differentially expressed genes that are closely immune‐related upon mild heat, accompanied by IL‐6 upregulation, a pyrogenic cytokine usually found during fever. When treated with intratumor PCN injection enabling mild heating in the tumor region, the 4T1 tumor‐bearing mice exhibit significantly improved antitumor immune effects compared with the control group. Superb efficacy is evident from pronounced apoptotic cell death, activated innate immune cells, enhanced tumor perfusion, and intensified innate and adaptive immune responses. This work highlights the crucial role of mild heat in modulating the microenvironment in optimum for improved immunotherapy, by converting the tumor into an in situ vaccine.

## 1 Introduction

Despite some technical breakthroughs in cancer treatment, reliable cure is still limited within the conventional methods such as surgery, chemotherapy, and radiotherapy. Among several novel cancer therapeutics, immunotherapy has recently been the center of attention in both basic medical science and clinical research communities, for its effectiveness, specificity, and adaptation to highly variant disease conditions.^1^, ^2^ The major immunotherapeutic approaches include checkpoint molecules programmed cell death 1 (PD‐1), programmed cell death ligand 1 (PD‐L1), and cytotoxic T‐lymphocyte antigen 4 (CTLA4). Immunotherapy adjuvant with CpG as Toll‐like receptor (TLR) agonists is capable of activating an innate immune response to potentiate vaccine‐specific immunity.^3^, ^4^ More than 100 clinical trials have been carried out involving CpG oligodeoxynucleotides (CpG ODN) as adjuvants and shown to be clinically effective.^3^ However, the drawback of CpG ODN‐based immunotherapy is the short‐termed benefits in patients, therefore not particularly viable and useful in a clinic setting.^4^ The inadequate efficacy is likely associated with the tumor microenvironment being detrimental to the immune system.^3^ The key to address this critical issue is, therefore, to modify the microenvironment toward immune activation that effectively enhances immunotherapy.

Fever, a typical symptom associated with infection and inflammatory disease, has been identified as an operating mechanism responsible for improved survival and the resolution of many infections like cancer.^5^ For instance, cancer immunotherapy by so‐called Coley toxins has shown direct correlation between survival rate and fever, in which 60% of the patients, who experienced fever, survived after five years.^6^, ^7^, ^8^ Benefits of fever have been found to enhance immunological functions of the organism almost at every step of innate and adaptive immunity including: regulation of pyrogenic cytokines IL‐6, recruitment and cytotoxic activity of immune cells, elevated antigen presentation capacity, and improved lymphocyte trafficking across high endothelial venules.^5^ Even with the above advantages, the uncontrolled and negative systematic outcomes, including sepsis or neurological injuries, have impeded the translation of Coley toxins into main clinical intervention of cancer.^9^ Nonetheless, it has inspired us to propose a hypothesis: the local hyperthermia relevant with fever may potentiate CpG immunotherapy. To our knowledge, fever‐relevant temperature is still rarely considered as a critical variable in pursuit of improved immunotherapy.^10^, ^11^

To validate this hypothesis, we developed a photothermal CpG nanotherapeutics (PCN) to identify the critical role of mild heat in regulating tumor microenvironment. CpG was integrated with photoheat conversion agent Au nanorod (Au), assembled by ovalbumin (OVA) protein, a usual carrier protein in vaccine design, for PCN. The local temperature rise was achieved by intratumoral injection of PCN, by illuminating the tumor area with an near‐infrared (NIR) light. Upon intratumoral injection of PCN, the fever‐like temperature of 43 °C was achieved to induce an immunofavorable tumor microenvironment. In this fashion, the immune system was facilitated to suppress tumor via enhanced immunoefficiency of CpG‐based immunotherapy. Gene profile analysis was employed to identify the underlying mechanism of microenvironment regulation at the genetic level.

## 2 Results

### 2.1 Design, Self‐Assembly, and Characterization of PCN

**Figure 1:**
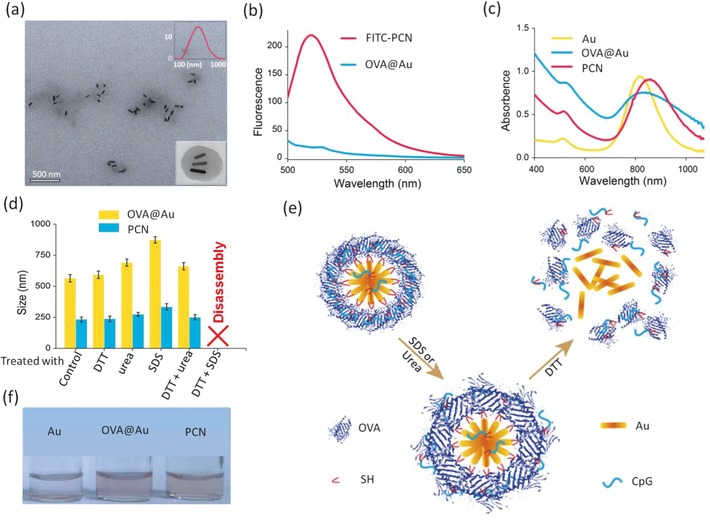
PCN characterizations. a) TEM image of PCN. The inset figure is the *z*‐average hydrodynamic diameter of PCN. b) Fluorescence spectra of FITC‐PCN and OVA@Au in water. c) UV–vis spectra of Au, OVA@Au, and PCN in water. d) The alteration of *z*‐average hydrodynamic diameters of PCN and OVA@Au in the presence of 30 × 10^−3^ m DTT, 1% SDS, and 8.0 m urea alone or in combinations. The PBS solution of PCN or OVA@Au without any perturbing reagents was used as the control. e) The schematic of the disassembly process of PCN. f) Photograph of Au, OVA@Au, and PCN in PBS.

Au nanorod with photoheat conversion efficiency^12^ was employed as the thermal agent, as well as the scaffold to integrate exterior immunoactive components. OVA protein was assembled with the Au nanorods via free thiols and disulfide bond bearing in the OVA protein structure to afford biocompatibility and biodispersion capacity. CpG was selectively integrated within the hybrid through interaction with OVA and coordination effect of terminal thiol group with Au nanorod. The entire hybrid PCN was developed by sonication which was supposed to facilitate the growth of OVA layer onto Au nanorods by disulfide bond cross‐linking. The average size of PCN is around 200 nm as characterized by dynamic light scattering (DLS) and transmission electron microscopy (TEM) (**Figure** **1**a). As investigated by TEM, OVA layer with light contrast could be clearly observed in individual PCN nanostructure, which encompasses several Au nanorods due to fusion of interparticles. Fluorescence of FITC‐labeled CpG exhibits a strong emission peak at 520 nm indicating effective loading of CpG in the nanosystem as shown in Figure 1b. Figure S1 (Supporting Information) also shows the retarded CpG mobility of PCN based on agarose gel electrophoretic analysis. CpG incorporation results in a size decrease of PCN compared with OVA@Au whose average size is 570 nm, leading to a more compact nanostructure showing it helps to stabilize the hybrid nanosystems and prevent aggregation (Figure S2, Supporting Information). Compared with Au nanorod, both the transverse and longitudinal peaks of PCN experience redshift (Figure 1c), which is ascribed to the plasmonic coupling of Au nanorod in the nanosystem as investigated by a TEM study.^13^ As shown in Figure 1f, PCN is well monodispersed in PBS overnight, whereas the “bare” Au nanorod quickly precipitates upon addition of saline.

To confirm the role of disulfide bond in PCN assembly, dissociation reagent dithiothreitol (DTT) was added to treat the solution of PCN and OVA@Au. As shown in Figure 1d, the hydrodynamic diameters of PCN do not experience obvious changes when treated with DTT alone, which deviates from the expectation. Therefore, other perturbing reagents like sodium dodecyl sulfate (SDS), and urea, were used to disrupt hydrophobic interactions, and hydrogen bonds, respectively. The size of PCN only increases slightly in the presence of SDS or urea, indicating a bit of swelling but with the intact nanostructure. Surprisingly, PCN disassembles entirely when treated with DTT and SDS, again with tiny swelling if treated with urea and SDS. These results indicate the formation of PCN in which the Au nanorod is surrounded by a layer of OVA and CpG molecules linked by disulfide bond and hydrophobic interaction. Existence of hydrophobic interaction is probably resulted by sonication mediated OVA denaturation which exposes the hydrophobic regions. The schematic of the disassembly process of PCN is shown in Figure 1e.

**Figure 2:**
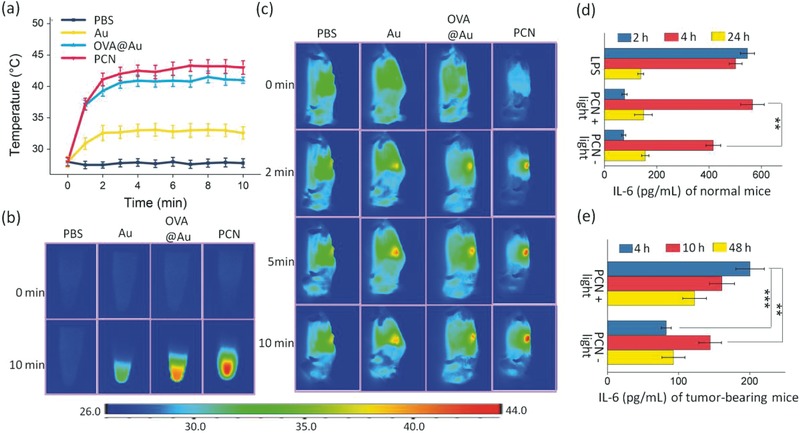
a–c) Photothermal property of PCN and d,e) mild heat induced enhanced IL‐6 expression. a) Temperature increase curves of PBS, Au, OVA@Au, and PCN in PBS dispersion with the NIR laser (808 nm, 0.5 W cm^−2^) irradiation for 10 min. b) In vitro infrared thermal images before and immediately after illumination. c) In vivo infrared thermal images at various time points. d) The level of IL‐6 in the serum of normal mice at various time points after stimulation. e) The level of IL‐6 in the serum of tumor‐bearing mice at various time points after stimulation. The data are presented as means ± s.d. (*n* = 6). Statistical analysis was performed by one‐way factorial ANOVA. The differences among groups were further analyzed by the post hoc multiple comparisons test (Student–Newman–Keuls). * *p* < 0.05, ** *p* < 0.01, *** *p* < 0.001.

The photothermal property of PCN was studied using an 808 nm laser. A short irradiation (2 min) elevates temperature of PCN or OVA@Au solutions to around 40 °C (**Figure** **2**a). Under the same condition, Au nanorod solution without OVA layer exhibits a modest temperature increase up to 31 °C. It should be noted that CpG incorporation is advantageous for photothermal efficiency, due to smaller size of PCN thus higher density of Au nanorod for stronger plasmonic coupling effect.^13^ Figure 2b shows the thermal images of PCN with superior photothermal conversion efficiency over both Au nanorod and OVA@Au. After intratumoral injection into tumor‐bearing mice, the infrared thermal images of the tumor region of mice treated with PBS, Au nanorod, OVA@Au, and PCN, irradiated by an 808 nm laser, was captured, as shown in Figure 2c. A pronounced advantage in temperature increasing rate was observed for PCN with the highest photothermal conversion efficiency. The in vivo thermal effect of Au nanorod, OVA@Au is more obvious compared with in vitro experiment, probably associated with tendency to aggregation in PBS compared with PCN. This tendency would be reduced in vivo for tissue barrier. The result reveals the synergistic effect of triple components in nanoassembly process. The tumor regions treated with PBS shows only a slight temperature increase (about 2 °C). An additional note is only a local heating effect without significant temperature changes in the surrounding tissues was found, beneficial to avoid the off‐target injury.

### 2.2 Mild Heat Induced and Increased IL‐6 Expression

The pyrogenic cytokine IL‐6 which is generated by infection stimulated macrophages and dendritic cells (DCs) is an important cytokine relevant with fever, both in the induction of temperature rise and the downstream mediation of lymphocyte trafficking to lymphoid organs and tumors.^5^ To investigate a fever‐like symptom by mild heat, we compared the level of IL‐6 among three groups: (1) normal mice treated with PCN without light, (2) normal mice treated with PCN‐based light stimulation, and (3) normal mice treated with lipopolysaccharides (LPS) as the positive group. LPS as a component of Gram‐negative bacteria cell walls was used to model immune‐induced fever response.^14^ IL‐6 expression at 2, 4, and 24 h post‐treatment was assessed. As shown in Figure 2d, the serum IL‐6 level of normal mice treated with mild heat via PCN reaches the highest at 4 h post‐treatment with comparable expression levels of LPS, though LPS induces quicker response that peaked at 2 h. Without light irradiation, the peaked IL‐6 expression is obviously lower than both light group and LPS group. At 24 h after stimulation, IL‐6 expression tends to be back to normal among all of the three groups, which is essential to reduce the potential risk of the sustainable IL‐6 induction. Merit of easy control of light also provides a convenient and fast way to control IL‐6 induction. In order to further verify this fever‐like symptom on tumor‐bearing mice, the serum IL‐6 level of PCN and light‐treated PCN group was compared post‐treatment. Again, the serum IL‐6 of the light‐treated PCN group shows a higher level after stimulation (Figure 2e). This potentially points to an improved immunotherapy effect by an IL‐6 trans‐signaling regulated lymphocyte trafficking mechanism, a condition similar to fever.

### 2.3 Mild Heat Induced Immune‐Associated Gene Profile Alterations

**Figure 3:**
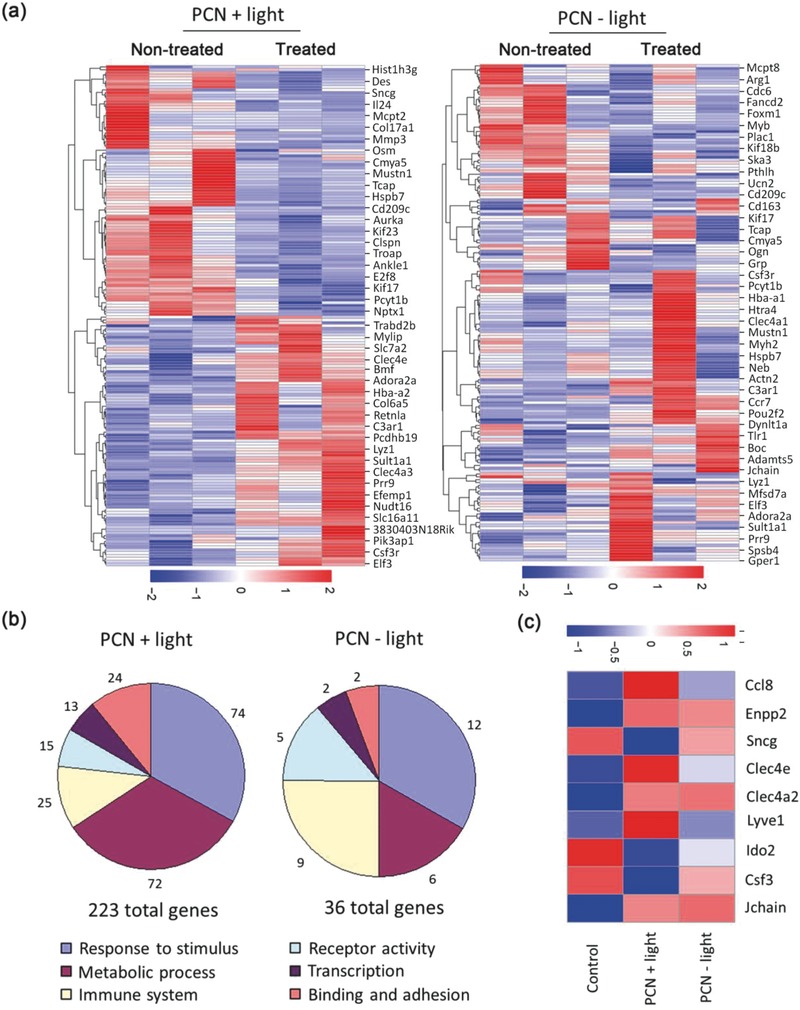
Altered tumor genetic profile in response to local therapy treatment. a) Differential gene expression heat maps of mice treated with PCN with and without light. b) Pathway analysis of significantly altered genes after different treatments. c) Selection of immune‐related DEGs that belong to light‐treated group. The whole‐genome data are representative from three different tumors.

Illumina platform was used to compare changes in gene expression profiles of the PCN‐injected tumors with and without laser irradiation. A clustergram of genes that are expressed following the treatment of mice was presented in **Figure** **3**a. To define differentially expressed genes (DEGs), an absolute fold change of ≥1.5 with an adjusted *p*‐value of <0.05 was used. According to this definition, the number of DEGs between the control group and the PCN‐based mild heated group (with light irradiation), is 146 (including 67 upregulated and 79 downregulated). For the correlation between the control group and the PCN‐treated group (without light irradiation), the number of DEGs is 35. The volcano plots for different treatments are developed by plotting the negative log of the *p*‐value on the *y*‐axis. Up‐ and downregulated genes are represented by red and green dots, respectively (Figure S3, Supporting Information).

To investigate the possible biologic functions of the transcript profiling data, the above described DEGs were analyzed by gene ontology (GO). Partial typical biological process, cellular component, and molecular function are presented in Figure S4 (Supporting Information). The significantly over‐represented GO terms are shown in Figure 3b. The GO terms of the altered genes for the light‐treated group are mainly related to binding and adhesion, responsive to stimulus, metabolic process, immune system, receptor activity, and transcription.

In this study, we focused our attention on the immune‐related DEGs for the light‐treated PCN group. Nine immune‐related DEGs were found to be differentially expressed due to modulation of light, among which there are six upregulated and three downregulated genes (Figure 3c). Of the six upregulated genes, two (CCL8 and Clec4e) are involved mainly in the attraction and adhesion of immunocyte. The highly upregulated gene, CCL8, can attract multiple biomolecules and cells including monocytes, T lymphocytes, eosinophils, basophils, NK and DC cells. In antitumor studies, CCL8 was reported to inhibit tumor growth and metastasis mainly through recruiting monocytes and T lymphocytes into the tumor area.^15^, ^16^, ^17^ Clec4e, a C‐type lectin receptor expressed on the cell surface of activated macrophages is strongly upregulated by a variety of inflammatory stimuli, including injury and irritation.^18^ Clec4e facilitates production of inflammatory cytokines and chemokines, and enhances subsequent recruitment of neutrophils to sites of inflammation.^19^ Another upregulated gene, lymphatic vessel endothelial hyaluronan receptor 1 (LYVE‐1), is expressed in lymphatic endothelium, blood sinus endothelium, and certain macrophage lineages. LYVE‐1‐expressing cells are likely involved in the uptake of foreign particles,^20^ presumably associated with improved lymph transportation.

Three downregulated genes (SNCG, IDO2, and Csf3) are reported to be immunosuppressively responsible for activation and proliferation of the main effector cells in cell‐mediated immunity. SNCG which contributes to immunosuppressive effects via the inhibition of DC differentiation and activation is shown to promote cell growth and motility leading to cancer metastasis and invasiveness.^21^ Indoleamine 2,3‐dioxygenase 2 (IDO2), a newly discovered enzyme, is able to catabolize tryptophan into kynurenine, therefore highly expressed in various types of cancer cells, as well as immune cells. IDO2 is a known inhibitor to T cell proliferation, and thus essential for regulatory T cell generation in healthy conditions. IDO2 silencing in tumor cells can delay tumor formation and arrest tumor growth in vivo.^22^ Colony stimulating factor 3 (Csf3), also known as granulocyte colony‐stimulating factor (G‐CSF), has been shown to influence various types of innate and adaptive immune cells. Csf3 is also known to affect the function of cytotoxic CD8^+^ T cells and T cell activation.^23^

### 2.4 Mild Heat Mediated Immunofavorable Response

We studied the mild heat mediated immunofavorable response in terms of tumor cell apoptosis, recruitment and activation of the innate immune cells, tumor perfusion, and innate immune response.

#### 2.4.1 Mild Heat Enhanced Tumor Cell Apoptosis

**Figure 4:**
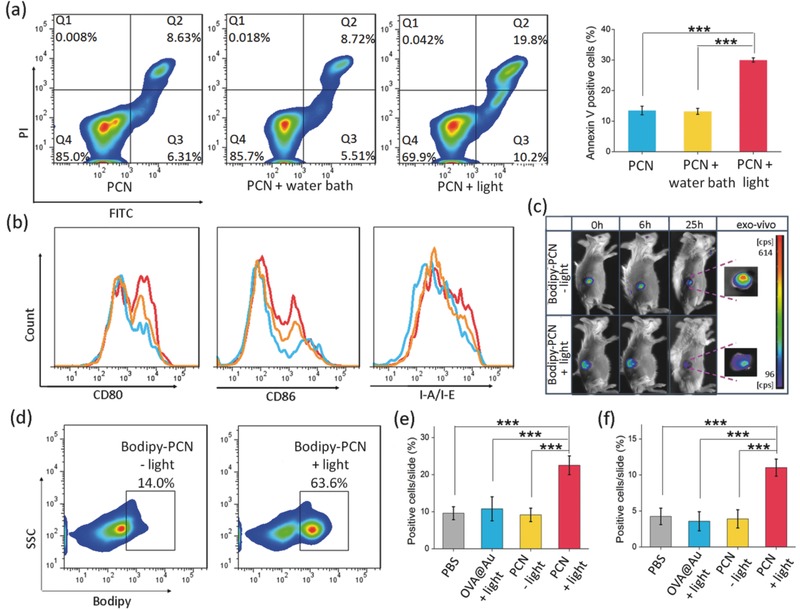
Mild heat mediated immunofavorable responses. a) Apoptosis in PCN‐treated 4T1 cells. 4T1 cells were heated to 43 °C for 10 min using an 808 nm laser or a water bath. The apoptosis of 4T1 cells after 24 h of hyperthermia treatment was analyzed using Annexin‐V assay. b) In vitro expression of CD80, CD86, and I‐A/I‐E of bone marrow‐derived DCs by flow cytometry after different treatments (untreated, PCN − light treated and PCN + light treated BMDCs were represented by blue lines, yellow lines, and red lines, respectively). c) Variation of bodipy‐labeled PCN in the tumor area with and without photothermal treatment by a live imaging system. d) Examination of bodipy content in TDLN at 48 h after treatment by flow cytometric analysis. Immunohistochemical analysis of tumors infiltrated by e) immune cells CD45^+^ leukocytes and f) F4/80^+^ macrophages after different treatments (The data are represented by percent of positive cells/total number of cells per slide). For (a), (e), and (f), the data are presented as means ± s.d. (*n* = 3). Statistical analysis was performed by one‐way factorial ANOVA, * *p* < 0.05, ** *p* < 0.01, *** *p* < 0.001.

In this study, PCN is able to generate in situ tumor associated antigens by inducing the apoptosis and necrosis of tumor cells after illumination. The apoptotic efficacy induced by mild heat in 4T1 cells (water bath hyperthermia used as the control) was evaluated by an apoptosis assay kit. 10 min irradiation significantly increases the percentage of apoptotic cells (as high as 30.0%), twofold compared with that of the water bath treatment and 37 °C control (**Figure** **4**a), likely associated with the different thermal models. With light irradiation, PCNs disperse within the matrix of cells can generate a temperature gradient between the neighbor cells. These cells nearby may experience local temperatures higher than the average temperature, therefore the susceptibility of 4T1 cells is increased to apoptosis. The cells heated with water, however, are only affected by the bulk temperature rise. Thus, the in situ tumor inhibition by mild hyperthermia may induce a more intense immune response with more tumor antigen epitopes with immune adjuvant CpG.

#### 2.4.2 Mild Heat Increased Activation of Innate Immune Cell

It is well‐known that DCs, as professional antigen presenting cells (APCs), play pivotal roles in the initiation and regulation of innate and adaptive immunities. The activation and maturation of DCs is a critical factor that determines the quality of the adaptive immunities.^24^ We studied the effect of mild heat on the maturation of bone marrow‐derived DCs (BMDCs) by the maturation markers: CD80, CD86, and I‐A/I‐E. It is found that the light‐treated PCN group obviously upregulated the expression of CD80, CD86, and I‐A/I‐E (Figure 4b), thus promoted the activation and maturation of BMDCs.

Secretion of proinflammatory cytokines is one of the important characteristics of immunocyte after activation. IL‐6 is a proinflammatory cytokine secreted by macrophages and dendritic cells to stimulate immune responses to trauma, causing tissue damage like inflammation.^25^ Thus, the aforementioned higher IL‐6 level upon mild hyperthermia is probably an important input for increased activation of innate immune cell.

#### 2.4.3 Mild Heat Enhanced Tumor Perfusion

Tumor perfusion, which is highly associated with the effect of immunotherapy, can render the pre‐existing antitumor response ineffective by blocking tumor penetration through stroma or retention of immune cells in the stroma.^26^ It is found that local heating is often associated with enhanced perfusion.^27^ The influence of mild heat on the perfusion of tumor via diffusion of bodipy‐labeled PCN was investigated by a live imaging system. As shown in Figure 4c, the fluorescent intensity of the light‐treated PCN group gradually decreases as a function of time. It was found that the light‐treated PCN group was hardly detected at 25 h post‐treatment, while considerable retention remained within tumors of the group without light. At 48 h after treatment, tumors were sectioned and imaged for comparison. Ex vivo fluorescent images showed an obviously reduced fluorescence intensity of the light‐treated PCN group, which may be caused by the enhanced physical tumor perfusion or by transportation with the help of immune cells. Mild heat treatment and CpG administration could activate DC and macrophages to capture the nanomaterials and traffic to the tumor‐draining lymph node (TDLN), for temperature‐dependent DC engulfment and migration activity.^28^, ^29^ Thus, portions of the PCN can be engulfed by APCs and then trafficked to TDLN. Analysis of bodipy fluorescence in TDLN by flow cytometric analysis shows a much high content (63.6%) of bodipy in the light‐treated PCN group, while only 14.0% positive signals in the PCN group without light (Figure 4d). These results indicate that mild heat of tumor tissues can further provide immune‐mediated benefit.

#### 2.4.4 Mild Heat Enhanced Innate Immune Response

Accompanied with the increased tumor antigen exposure and enhanced vascular perfusion, a more intense innate immune response was induced as demonstrated by the immunohistochemical analysis of tumors 2 d after treatment. The presence of tumor‐infiltrating CD45^+^ leukocytes and F4/80^+^ macrophage is significantly higher in light‐treated PCN group than in those treated with PCN only and the control (Figure 4e,f, and Figure S5, Supporting Information). These results show that mild heat can intensify the inflammatory response of PCN that leads to recruit more immunological cells to tumor.

### 2.5 Mild Heat Improved Immunotherapy Effect

**Figure 5:**
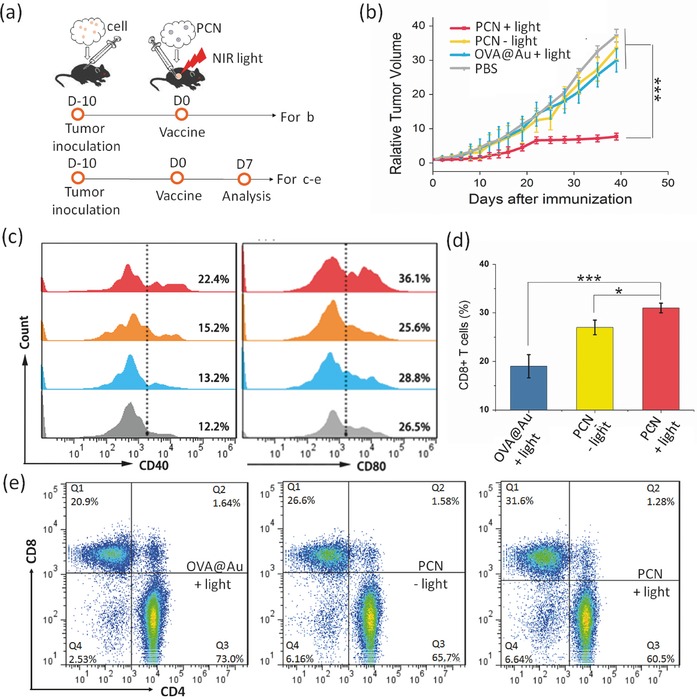
Mild heat improved immunotherapy effect and adaptive immune response. a) Schematic illustration of the animal experiment. b) Relative tumor growth curves of mice in different groups. The experiments were repeated for three independent experiments. c) In vivo expression of CD40 and CD80 of DC in the draining lymph node by flow cytometry 7 d after different treatments. d,e) Proportion of spleen‐infiltrating CD8^+^ T cells and CD4^+^ T cells analyzed by flow cytometry for T cell infiltration (gated on CD3^+^ T cells). For (b), (d), and (e), the data are presented as means ± s.d. (*n* = 5). Statistical analysis was performed by one‐way factorial ANOVA, * *p* < 0.05, ** *p* < 0.01, *** *p* < 0.001.

Induced by the immunofavorable tumor microenvironment after mild heat, immune therapy effect was assayed using 4T1 tumor‐bearing mice as the model which shares similar phenotypes of immune‐suppressive microenvironment with the clinical human mammary cancers.^30^, ^31^ Mice were randomly divided into four groups. One group was intratumorally injected with PCN and irradiated with an 808 nm laser for 10 min, with temperature of tumor region controlled at 43 °C during irradiation. As shown in **Figure** **5**b, tumor growth of the light‐treated PCN group is largely suppressed as compared to that of PCN group without light and the control group. Notably, the tumors of four mice (five mice in each group) completely disappear at day 15th after treatment (Figure S6a, Supporting Information). The mice of light‐treated PCN group also have prolonged survival as shown in Figure S6b (Supporting Information). To further evaluate the enhanced anti‐tumor immunity of mild heat, a rechallenge model was established in which mice had one tumor first and were inoculated with a secondary tumor one week later. Mice in light‐treated PCN group show an obviously delay in rejection of the secondary rechallenge (Figure S6c, Supporting Information).

### 2.6 Mild Heat Enhanced Adaptive Immune Response

To identify the underlying mechanism of tumor inhibition by PCN‐based mild heat, the maturation status of DCs in the DLN, the major cytokines in the serum and CD8^+^ T lymphocytes (CD8^+^ CTLs) in the spleen and tumor were analyzed one week after immunization. Activation of innate immune system is only the first step in the process of antitumor immune response. The main function of the innate immune cell, especially macrophages and DCs, is to activate the most important cytotoxic CD8^+^ CTLs in antitumor immunology by uptaking the tumor‐specific antigens in the innate immune stage. As one of the most important classes of antigen presenting cells, DCs only exert their antigen presenting ability when transformed from immature to mature state during the antigen processing and migration process. The maturation status of DCs within DLN was assessed. As shown in Figure 5c, light treatment of tumors after injection of PCN greatly promotes maturation of DCs in vivo. DCs upon maturation secrete various types of cytokines to regulate other types of immune cells.^32^ Thus, two major cytokines, namely, interleukin 12 (IL‐12) and interleukin 1β (IL‐1β) secreted by DCs were analyzed by ELISA. The ELISA analysis was also performed for the fever‐associated pyrogenic cytokine IL‐6 in the mouse sera after different treatments. Mice whose tumors treated with light after injection of PCN show higher levels of IL‐6, IL‐12, and IL‐1β than those without light (Figure S6d–f, Supporting Information). Activated by DCs, more CD8^+^ CTLs are observed in spleen and the tumors treated with light after the injection of PCN (Figure 5d,e and Figure S7, Supporting Information).

## 3 Discussion

**Figure 6:**
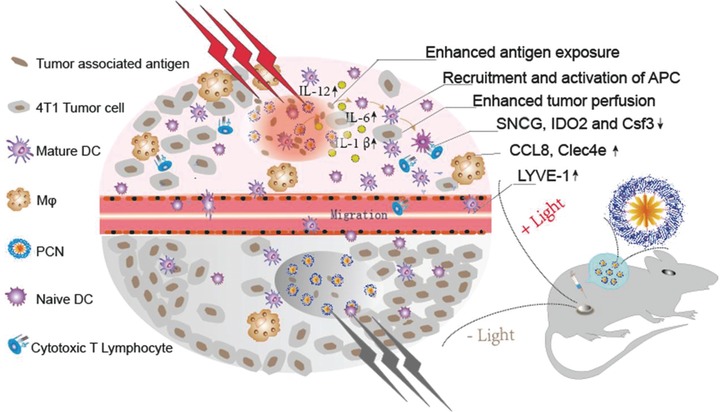
An immunofavorable tumor microenvironment established via fever‐like immunoresponse induced by the photothermal effect of PCN. Upon mild heat, two upregulated genes (CCL8 and Clec4e) are involved mainly in the attraction and adhesion of immunocyte. Three downregulated genes (SNCG, IDO2, and Csf3) are responsible for activation and proliferation of the main effector cells in cell‐mediated immunity. This is accompanied by increased antigen exposure, recruitment and activation of the innate immune cells, enhanced tumor perfusion, and intensified immune response.

CpG has been shown to induce a Th1‐biased immune response and support CD8^+^ T cell responses, enabling them to be the promising adjuvants for cancer vaccines.^33^, ^34^, ^35^ However, the tumor elimination efficiency of CpG‐based vaccines is usually counteracted by the immunosuppressive microenvironment of the established tumors. It is, therefore, critical to develop a new approach that offsets the original balance and establishes a microenvironment favorable to the tumoricidal activity. An immunofavorable tumor microenvironment can be established via fever‐like immunoresponse induced by the photothermal effect of PCN (**Figure** **6**).

Tumor cells are more sensitive to local hyperthermia than normal cells. It is reported that temperatures over 42.5 °C is cytotoxic to tumor cells,^36^ and temperatures exceeding 50 °C can be used to achieve significantly improved cancer therapy in combination with immune adjuvants and immune checkpoints.^37^, ^38^, ^39^, ^40^, ^41^, ^42^ The above efforts provided new insights on the development of novel techniques for cancer treatment. From immune therapeutic aspect, a high ablation temperature may be detrimental to local anticancer immune cells and microenvironment. For example, while 43 °C might be favorable for vasculature permeability and facilitating trafficking of DCs between tumors and DLN, even slightly higher temperature at 45 °C can be harmful to vasculature.^43^ Moreover, the latest report on photothermal therapy for cancer immunotherapy treatment suggested that mild hyperthermia (42 °C) could minimize the potential thermal‐induced denaturation of the tumor antigen and other biomolecules.^11^ Bearing this in mind, we hypothesize a mild hyperthermia relevant with fever temperature in the tumor area would be favorable. To this end, PCN as the unique platform was developed to integrate photothermal property as well as efficient CpG encapsulation. An apoptosis assay shows that upon a mild heat by illumination PCN could significantly mediate tumor cell apoptosis and necrosis. This is particularly favorable for tumor antigen release^44^, ^45^, ^46^ so as to associate with CpG payloaded in PCN synergistically to exert an antitumor immune therapy effect.

Once sensing antigens and CpG, the local innate immune cells, including macrophages and DCs, are activated to produce proinflammatory cytokines and recruit more APCs. The mild local hyperthermia in the tumor area further amplifies the immune response by these successive innate immune stages which consequently promote activation/maturation of innate immune cells. This is well demonstrated by the upregulated expression of BMDC maturation markers, the increased serum IL‐6 level, and the upregulated gene expression profiles of CCL8 and Clec4e which is mainly responsible for attraction and adhesion of immunocyte of the light‐treated PCN group. Evoked by the proinflammatory cytokines and chemokine secreted by the local immune cells, APC‐like macrophages and DCs are recruited to tumor in the successive stage. Additionally, it is found that mild local hyperthermia in the tumor area has a pronounced effect on the tumor microenvironment by enhanced tumor perfusion and trafficking of immunocyte between tumors and peripheral immune organs. An increased number of tumor‐infiltrating leukocytes and macrophages are observed in tumor. The enhanced vascular perfusion also leads to accelerated transportation of bodipy‐labeled nanomaterial from tumor to TDLN. Therefore, homing of APCs to TDLN has also been accelerated by mild hyperthermia in the tumor area.

After relocating to peripheral immune organs, APCs present the tumor‐associated antigens to T cells and drive the population expansion of tumor‐specific effector CD8^+^ T cell. As a result of enhanced innate immune response via mild local hyperthermia, the number of CD8^+^ T cell is effectively increased in spleen. Following activation and proliferation, the cytotoxic CD8^+^ T lymphocytes need to traffic into tumors to attack cells that express relevant antigens. However, vascular barriers must be crossed allowing for intensive CD8^+^ T cell accumulation.^47^, ^48^, ^49^, ^50^ The higher level of IL‐6 upon mild heat treatment is essential in promoting lymphocyte trafficking to tumors. Low migration of CD8^+^ T cells into the tumor microenvironment has been attributed to the limited availability of trafficking molecules ICAM‐1 in tumor vessels.^51^ An IL‐6 trans‐signaling mechanism has been identified for upregulating the biosynthesis of ICAM‐1 in tumor vessels. Therefore, a higher level of IL‐6 is favorable for accumulation of CD8^+^ T cells in tumor. This is consistent with our experimental results that a large amount of CD8^+^ T lymphocytes was found in the light‐treated PCN group with a high IL‐6 expression. Moreover, mild heat also downregulated the expression of several immunosuppressive genes SNCG, IDO2, and Csf3 in the proliferation and activation of the CD8^+^ T cells. The antitumor immunity effect of the light‐treated PCN group was largely boosted by increasing of infiltration, proliferation, and activation of cytotoxic CD8^+^ T lymphocytes.

## 4 Conclusion

A highly versatile, integrative PCN nanosystem was developed by a convenient approach to coordinate with tumor microenvironment and reverse the tumor suppressive condition. This study highlighted the critical role of fever‐like heat in an immunofavorable microenvironment that significantly improved the efficiency of CpG‐based immunotherapies. Analysis of genetic profile demonstrated immune‐related DEGs that closely correlated with tumor microenvironment including immune cell recruitment (CCL8) and trafficking (ICAM‐1). The fever‐inspired strategy has been shown successful to enhance the innate and adaptive immune response, a critical bioprocess in tumor immunotherapy. This unique approach is therefore directly converting the tumor into an in situ vaccine by creating a microenvironment favorable for the vaccine to exert its immune effect.

## 5 Experimental Section

*Materials*: Unless otherwise indicated, all chemicals were purchased from Sigma‐Aldrich. The thiol–CpG 1826(5′‐TCC ATG ACG TTC CTG ACG TT‐3′) was purchased from Sangon Biotech Ltd. (Shanghai, China). APC‐conjugated anti‐mouse CD11c, PE‐conjugated anti‐mouse CD86, PE‐conjugated anti‐mouse CD40, FITC‐conjugated anti‐mouse CD80, FITC‐conjugated anti‐mouse I‐A/I‐E, APC‐conjugated CD3, FITC/Percp Cy5.5‐conjugated anti‐mouse CD8a, efluor 450‐conjugated anti‐mouse CD4, and purified anti‐mouse CD16/CD32 antibodies against cell surface markers for flow cytometry assay and ELISA kits were purchased from eBioscience (San Diego, CA). Fetal bovine serum (FBS), Dulbecco's modified Eagle's medium (DMEM), penicillin‐streptomycin, trypsin, and Dulbecco's phosphate‐buffered saline (DPBS) were supplied by Gibco Invitrogen Corp. Co. Ltd. Annexin V‐FITC Apoptosis Detection Kit was obtained from Beyotime Institute of Biotechnology. Paraformaldehyde (4%) was obtained from DingGuo Chang Sheng Biotech.

*Preparation of the PCN Ternary Hybrids System*: Thiol–CpG 1826 was first subject to a reduction treatment according to protocols provided by manufacturer, to acquire CpG terminated with free thiol groups. A PBS solution of OVA (0.57 mL, 2.0 mg mL^−1^) was mixed with 0.09 mL PBS solution of CpG (1.0 mg mL^−1^). Under sonication, the mixture was added drop by drop to a gold nanorod (Au) solution (0.23 mL, 80 µg mL^−1^) in 4 min and sonicated for another 4 min. The Au solution was obtained by the seeded growth protocol according to the literature.^52^ To identify the successful loading of CpG in the nanosystem, FITC‐labeled CpG was used to obtain the ternary hybrids system. After sonication, an ultrafiltration treatment (Amicon ultra‐4 centrifugal filter devices with molecular weight cutoff of 10K) was used to remove the free CpG.

*Preparation of the OVA@Au Binary Hybrids System*: The OVA@Au binary hybrids system was obtained similar to the PCN ternary hybrids system without adding CpG. Briefly, a PBS solution of OVA (0.57 mL, 2.0 mg mL^−1^) was added drop by drop to the gold nanorod (Au) solution (0.23 mL, 80 µg mL^−1^).

*Characterization*: The size distribution of the hybrids systems was tested by TEM (Tecnai‐12 Bio‐Twin, FEI, Netherlands) and DLS (Malvern Instruments Ltd., Worcestershire, UK). UV–vis and fluorescence spectra were recorded on an UV–vis spectrophotometer (Varian Ltd., Hong Kong) and a Cary Eclipse Fluorescence Spectrophotometer (Agilent Technologies), respectively.

*Particle Size of PCN and OVA@Au in Various Solvents*: To explore the driving forces involved in the formation of the nanosystem, the *z*‐average hydrodynamic diameter of PCN and OVA@Au was determined in the presence of perturbing solvents 30 × 10^−3^ m DTT, 1% SDS, and 8.0 m urea alone or in combinations. Briefly, to a PBS solution of PCN or OVA@Au, an equal volume of perturbing solvent was added. After 2 h standing, the corresponding particle size of the solution was then measured. The PBS solution of PCN or OVA@Au without any perturbing solvent was used as the control.

*In Vitro Photothermal Effect*: A 50 µL amount of OVA@Au or PCN dispersed in PBS in a 250 µL tube was irradiated with an 808 nm laser at a power density of 0.5 W cm^−2^ for 10 min. The temperature increase and thermal images of the dispersions were recorded by an infrared thermal camera (DALI TECHNOLOGY, LT3‐P). PBS and Au nanorod were used as control treated in the same conditions.

*Apoptosis Assay*: 4T1 cells were cultivated in 48‐well plates for 24 h. After the addition of PCN, the water bath‐treated and light‐treated groups were heated to 43 °C for 10 min using an 808 nm laser or a water bath, respectively. After being replaced with fresh DMEM, cells were cultivated overnight, then trypsinized and collected for Annexin V/PI staining following the manufacturer's instructions. Briefly, cells were suspended with 195 µL Annexin V‐FITC binding buffer, and then stained directly with 5 µL Annexin V‐FITC and 10 µL of PI. After 10 min of incubation in the dark on ice, the cells were analyzed by flow cytometry (BD Biosciences).

*Mouse Tumor Model*: The tumor was grown by inoculating 100 µL of 4T1 cells with the concentration of 6 × 10^5^ cells in PBS into the right flank of each Balb/c mouse (female, six weeks old) under anesthesia. All experiments involving live animals were performed in compliance with the relevant laws and institutional guidelines of Tongji University.

*In Vivo Infrared Thermal Imaging*: Tumor model was established as described above. After the tumor volume reached about 50 mm^3^, 50 µL of OVA@Au or PCN prepared was injected directly into the tumor area. The tumor area was then irradiated with an 808 nm laser at a power density of 0.5 W cm^−2^ for 10 min. The thermal image of the whole mouse was recorded by the infrared thermal camera post injection. PBS and Au nanorod were used as control treated in the same conditions.

*Cytokine Detection*: For the detection of IL‐6 in the serum of normal mice, mice were divided into three groups with six mice in each group. The positive group mice were injected subcutaneously with 50 µL PCN and irradiated the skin at the injection position with 808 nm laser at 43 °C for 10 min. In the other two control groups, only 50 µL LPS (0.1 mg mL^−1^) or 50 µL PCN was injected subcutaneously without irradiation. The blood sample of mice was obtained from their eyes at various time points after treatment. Then serum was isolated and diluted for analysis with ELISA kits according to vendors' protocols. For the detection of IL‐6 in the serum of tumor‐bearing mice, tumor‐bearing mice were used instead of normal mice without the LPS group. And PCN was injected intratumorally.

*In Vivo and Ex Vivo Fluorescence Imaging*: Tumor‐bearing mice were obtained as described above. After the intratumoral injection of 50 µL bodipy‐PCN, mice were divided into two groups with three mice in each group. One group was irradiated with an 808 nm laser at 43 °C for 10 min. The temperature of the tumor area was monitored by the infrared thermal camera. The other group was used as control without irradiation. Then the variation of the fluorescent intensity of bodipy was monitored by fluorescence imaging using a Maestro In Vivo imaging system (excitation at 630 nm, emission at 780 nm, CRI, MA) at 6 and 25 h post‐injection. At 48 h post‐injection, mice were sacrificed and tumor were taken out and imaged.

*Immunohistochemistry*: Tumor‐bearing mice were obtained as described above. After the tumor volume reached about 50 mm^3^, mice were divided into four groups with three mice in each group. For the two irradiation group, 50 µL of OVA@Au or PCN prepared was injected directly into the tumor area. The tumor area was then irradiated with an 808 nm laser at 43 °C for 10 min. For the other two groups, only PBS or PCN was injected without irradiation. Tumors were resected 48 h following treatment, fixed and embedded into paraffin. The fixed organs were sliced, stained with rabbit anti‐F4/80 antibody and anti‐CD45 antibody, and then subjected to optical microscopy study.

*Immunotherapy*: Tumor‐bearing mice were obtained as described above. After the tumor volume reached about 50 mm^3^, mice were divided into four groups with five mice in each group. For the two irradiation groups, 50 µL of OVA@Au or PCN prepared was injected directly into the tumor area. The tumor area was then irradiated with an 808 nm laser at 43 °C for 10 min. For the other two groups, only PBS or PCN was injected without irradiation. Tumor size was measured every 2 d. Tumor volume was calculated from the following relationship: *V* = *WL* ^2^/2 (*W* means the longest diameter, *L* means the shortest diameter). To evaluate whether specific immunologic memory responses were generated in mice that bearing 4T1 tumor cells, the mice were rechallenged with 6 × 10^5^ 4T1 tumor cells in the other flank 8 d following the immunotherapy. The mice were examined every 2 d to evaluate tumor development.

*Flow Cytometry (FACS) Assay of Cell Surface Markers*: For in vitro experiments, BMDCs were separated from Balb/c mice according to the literature.^53^ For PCN + light group, BMDCs were heated to 41 °C by 808 nm laser for 6 min after the addition of PCN and then cultured in the incubator for another 20 h. For PCN group, BMDCs were cultured for 20 h after the addition of PCN without irradiation. Then antibodies to CD11C, CD80, CD86, and MHCII were used to stain the cells. Then stained cells were analyzed on a Calibur FACS instrument (BD), and analyzed using FlowJo software. For in vivo experiments, the DCs were obtained from the draining lymph node of mice 7 d after various treatments. For in vivo T lymphocytes detection, the spleen of mice was dissected 7 d after various treatments and stained with antibodies to CD3, CD4, and CD8.

*Sample Preparation for Gene Expression Analysis*: Tumor‐bearing mice were obtained as described above and divided into three groups with three mice in each group. One group was injected intratumoral with 50 µL PCN and irradiated with 808 nm laser at 43 °C for 10 min. One group was also injected intratumorally with 50 µL PCN but without irradiation. The other group with only intratumoral injection of 50 µL PBS was used as control. The tumor RNA was prepared at 6 h after irradiation (the irradiation group) or injection (the other two groups).

*Statistical Analysis*: All values in the present study were expressed as mean ± s.d. All animal studies were performed after randomization. The significance of differences between the groups was analyzed by one‐way analysis of variance (ANOVA) with SPSS 17.0 (SPSS Inc., USA). Significant differences between or among the groups are indicated by *P < 0.05, **P < 0.01, ***P < 0.001.

## Conflict of Interest

The authors declare no conflict of interest.

## Supporting information

Supplementary
