# Supplementary material for: Fever‐Inspired Immunotherapy Based on Photothermal CpG Nanotherapeutics: The Critical Role of Mild Heat in Regulating Tumor Microenvironment
Source: Adv Sci (Weinh). 2018 Mar 25;5(6):1700805. doi: 10.1002/advs.201700805 (PMC6010888; doi:10.1002/advs.201700805)
Supplement: Supplementary file 1 — Supplementary [file ADVS-5-1700805-s001.pdf]

Copyright WILEY-VCH Verlag GmbH & Co. KGaA, 69469 Weinheim, Germany, 2018.

ADVANCED SCIENCE
Open Access

Supporting Information

for *Adv. Sci.*, DOI: 10.1002/advs.201700805

Fever-Inspired Immunotherapy Based on Photothermal CpG Nanotherapeutics: The Critical Role of Mild Heat in Regulating Tumor Microenvironment

*Yan Li, Lianghua He, Haiqing Dong, Yiqiong Liu, Kun Wang, Ang Li, Tianbin Ren, Donglu Shi, and Yongyong Li**

Supporting Information

Fever-inspired Immunotherapy Based on Photothermal CpG Nanotherapeutics: The Critical Role of Mild Heat in Regulating Tumor Microenvironment

*Yan Li, Lianghua He, Haiqing Dong, Yiqiong Liu, Kun Wang, Ang Li, Tianbin Ren, Donglu Shi, Yongyong Li**.*

Y. Li, L. He, H. Dong, Y. Liu, Prof. Y. Li

Shanghai East Hospital, The Institute for Biomedical Engineering & Nano Science, Tongji University School of Medicine, Shanghai 200092, PR China

E-mail: yongyong_li@tongji.edu.cn.

K. Wang, Prof. T. Ren

School of Materials Science and Engineering, Tongji University, 4800 Caoan Road, Shanghai 201804, PR China

A. Li

School of Life Science and Technology, Tongji University, 1239 Siping Road, Shanghai 200092, PR China

D. Shi

The Materials Science & Engineering Program, Department of Mechanical & Materials Engineering, College of Engineering & Applied Science, University of Cincinnati, Cincinnati, OH45221, USA

Detailed information for gene expression sequencing

RNA quantification and qualification: RNA degradation and contamination was monitored on 1% agarose gels. RNA purity was checked using the NanoPhotometer® spectrophotometer (IMPLEN, CA, USA). RNA concentration was measured using Qubit® RNA Assay Kit in Qubit®2.0 Flurometer (Life Technologies, CA, USA). RNA integrity was assessed using the RNA Nano 6000 Assay Kit of the Agilent Bioanalyzer 2100 system (Agilent Technologies, CA, USA).

Library preparation for Transcriptome sequencing: A total amount of 1 μg RNA per sample was used as input material for the RNA sample preparations. Sequencing libraries were generated using NEBNext®Ultra™ RNA Library Prep Kit for Illumina® (NEB, USA) following manufacturer's recommendations and index codes were added to attribute sequences to each sample. Briefly, mRNA was purified from total RNA using poly-T oligo-attached magnetic beads. Fragmentation was carried out using divalent cations under elevated temperature in NEBNext First Strand Synthesis Reaction Buffer (5X). First strand cDNA was synthesized using random hexamer primer and M-MuLV Reverse Transcriptase (RNase H-). Second strand cDNA synthesis was subsequently performed using DNA Polymerase I and RNase H. Remaining overhangs were converted into blunt ends via exonuclease/polymerase activities. After adenylation of 3' ends of DNA fragments, NEBNext Adaptor with hairpin loop structure were ligated to prepare for hybridization. In order to select cDNA fragments of preferentially 200-250 bp in length, the library fragments were purified with AMPure XP system (Beckman Coulter, Beverly, USA). Then 3 μl USER Enzyme (NEB, USA) was used with size-selected, adaptor-ligated cDNA at 37°C for 15 min followed by 5 min at 95°C before PCR. Then PCR was performed with Phusion High-Fidelity DNA polymerase, Universal PCR primers and Index (X) Primer. At last, PCR products were purified (AMPure XP system) and library quality was assessed on the Agilent Bioanalyzer 2100 system.

Clustering and sequencing: The clustering of the index-coded samples was performed on a cBot Cluster Generation System using TruSeq PE Cluster Kit v4-cBot-HS (Illumia) according to the manufacturer's instructions. After cluster generation, the library preparations were sequenced on an Illumina Hiseq platform and paired-end reads were generated.

Detailed information for gene expression data analysis

Quality control: Raw data (raw reads) of fastq format were firstly processed through in-house perl scripts. In this step, clean data (clean reads) were obtained by removing reads containing adapter, reads containing ploy-N and low quality reads from raw data. At the same time, Q20, Q30, GC-content and sequence duplication level of the clean data were calculated. All the downstream analyses were based on clean data with high quality.

Comparative analysis: The adaptor sequences and low-quality sequence reads were removed from the data sets. Raw sequences were transformed into clean reads after data processing. These clean reads were then mapped to the reference genome sequence. Only reads with a perfect match or one mismatch were further analyzed and annotated based on the reference genome. Tophat2 tools soft were used to map with reference genome.

Gene functional annotation: Gene function was annotated based on the following databases: Nr (NCBI non-redundant protein sequences); Nt (NCBI non-redundant nucleotide sequences); Pfam (Protein family); KOG/COG (Clusters of Orthologous Groups of proteins); Swiss-Prot (A manually annotated and reviewed protein sequence database); KO (KEGG Ortholog database); GO (Gene Ontology).

Differential expression analysis:

For the samples with biological replicates:

Differential expression analysis of two conditions/groups was performed using the DESeq R package (1.10.1). DESeq provide statistical routines for determining differential

expression in digital gene expression data using a model based on the negative binomial distribution. The resulting P values were adjusted using the Benjamini and Hochberg's approach for controlling the false discovery rate. Genes with an adjusted P-value <0.05 found by DESeq were assigned as differentially expressed.

For the samples without biological replicates:

Prior to differential gene expression analysis, for each sequenced library, the read counts were adjusted by edgeR program package through one scaling normalized factor. Differential expression analysis of two samples was performed using the DEGseq (2010) R package. P value was adjusted using q value. Q value<0.005 & |log2 （fold change） |≥1 was set as the threshold for significantly differential expression.

GO enrichment analysis: Gene Ontology (GO) enrichment analysis of the differentially expressed genes (DEGs) was implemented by the GOseq R packages based Wallenius non-central hyper-geometric distribution , which can adjust for gene length bias in DEGs.

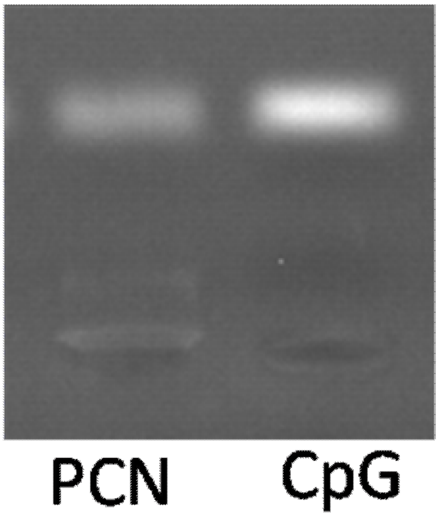

Figure S1. The agarose gel electrophoretic analysis of PCN compared with CpG. To guarantee the equal CpG amount, no ultra filtration treatment was performed after preparation of PCN.

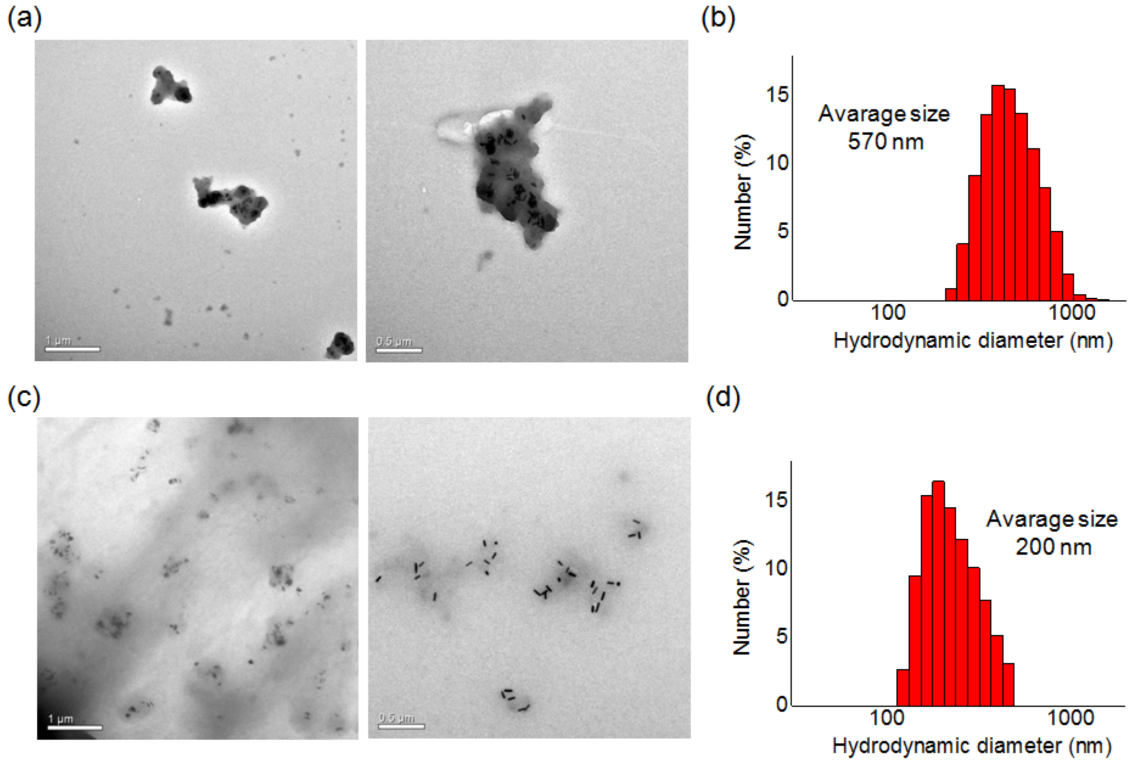

Figure S2. CpG incorporation strongly stabilizes the PCN nano-systems and effectively prevents aggregation. (a) TEM image of OVA@Au. (b) Hydrodynamic diameter distribution of OVA@Au. (c) TEM image of PCN. (d) Hydrodynamic diameter distribution of PCN. Compared with OVA@Au, CpG exhibits a significant effect on the stabilization of the hybrids nano-systems which could prevent the hybrids nano-systems PCN to form aggregation.

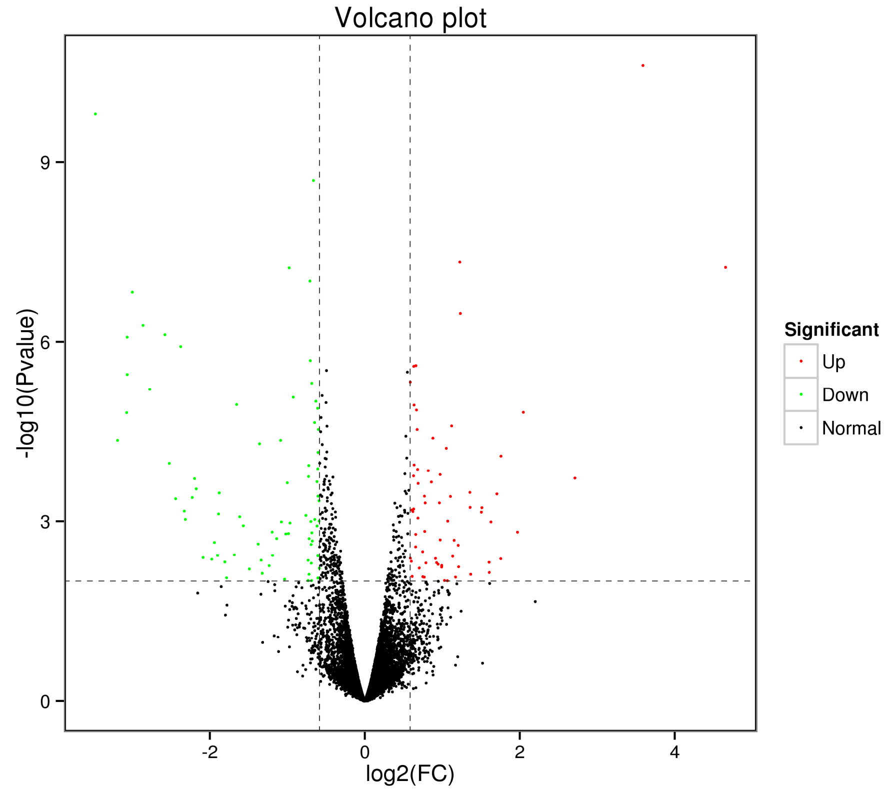

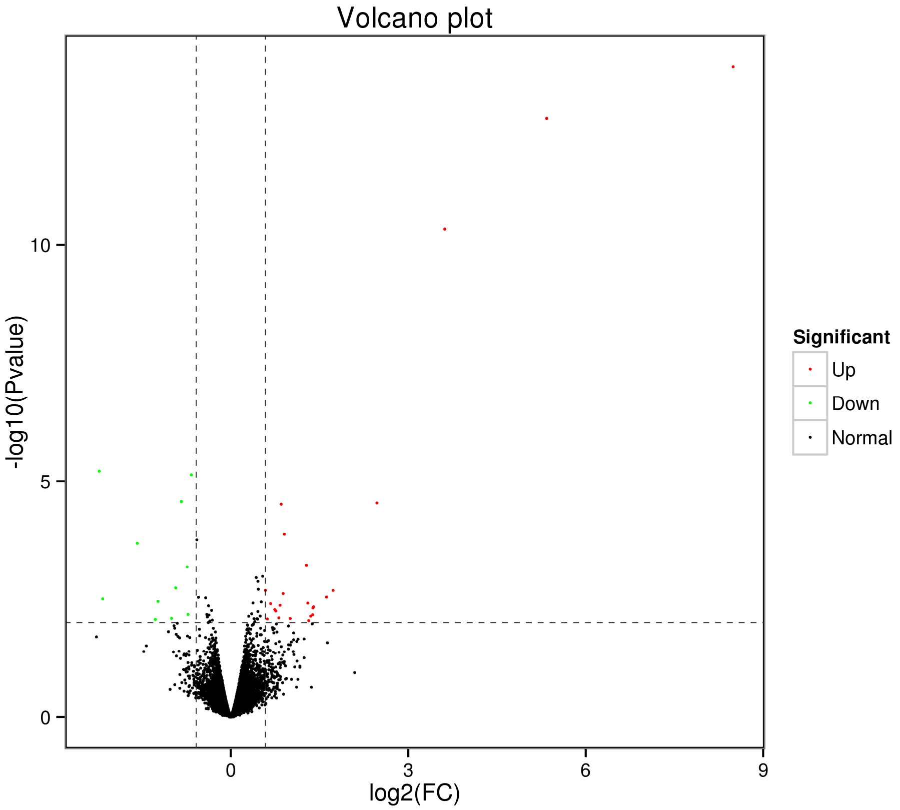

Figure S3. Volcano plots for the PCN injected tumor with (up) and without light (down).

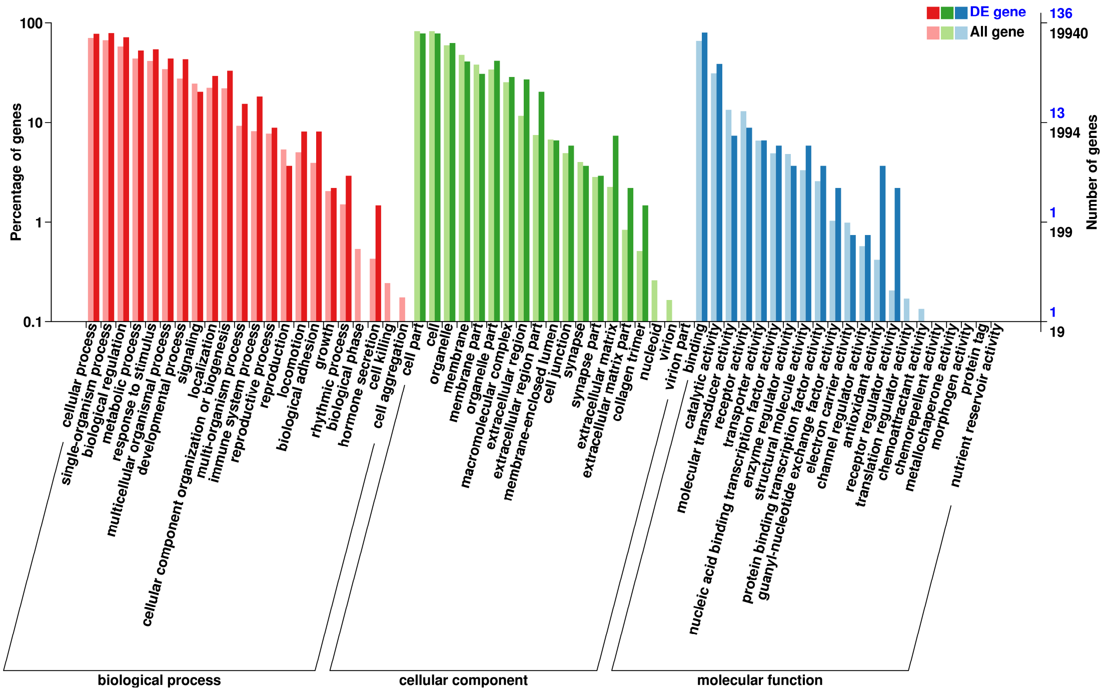

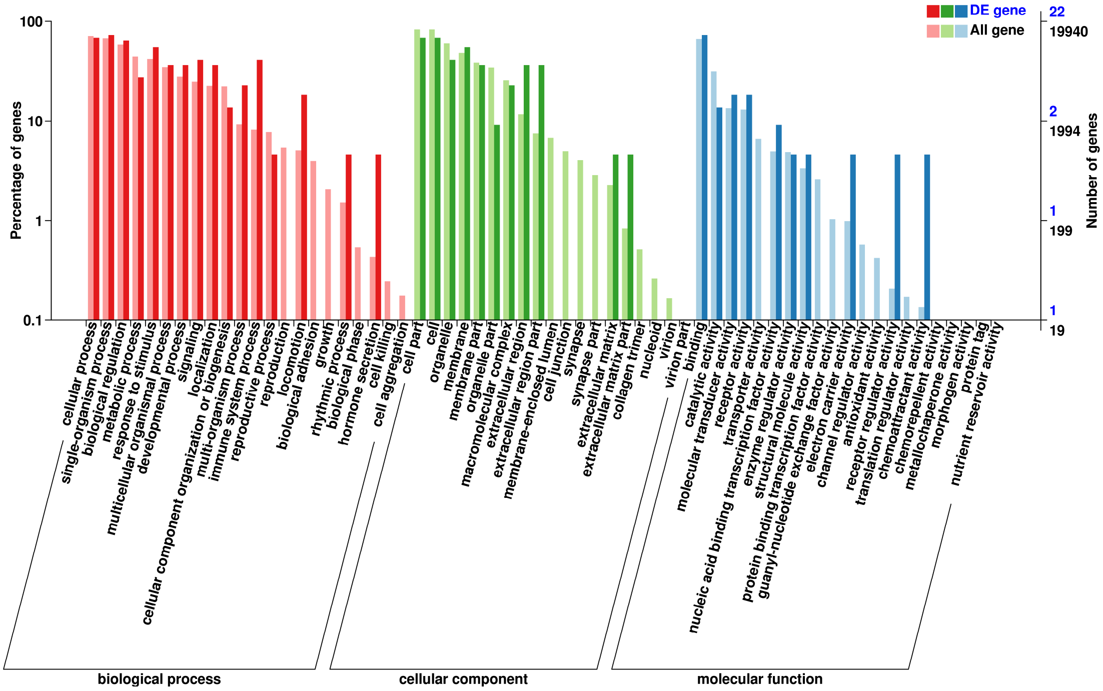

Figure S4. Histogram presentation of Gene Ontology classification. The results are summarized in three main categories: biological process, cellular component and molecular function.

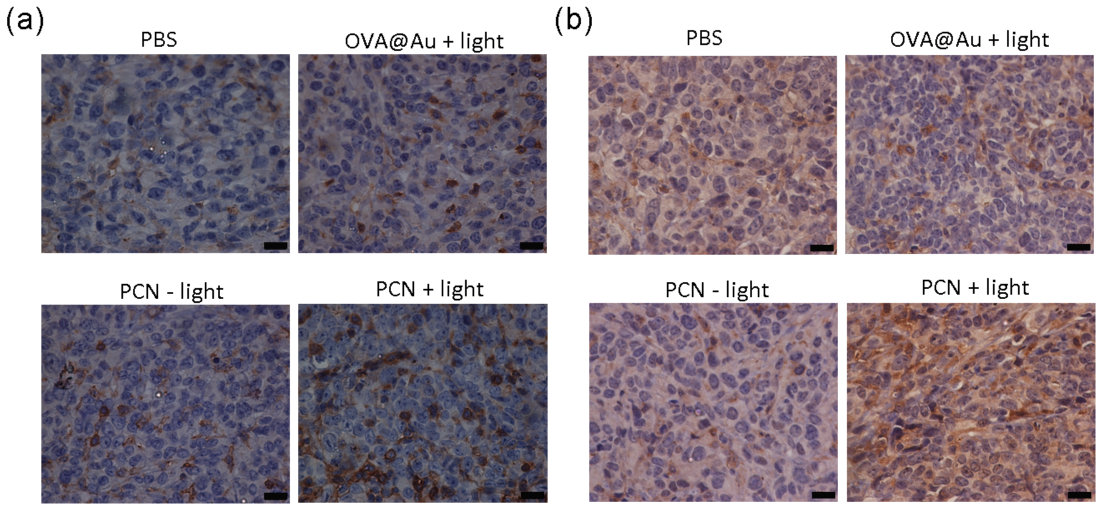

Figure S5. Immunohistochemical analysis of tumors infiltrated by immune cells CD45$^+$ leukocytes (a) and F4/80$^+$ macrophages (b) after different treatment. All scale bars are 100 μm.

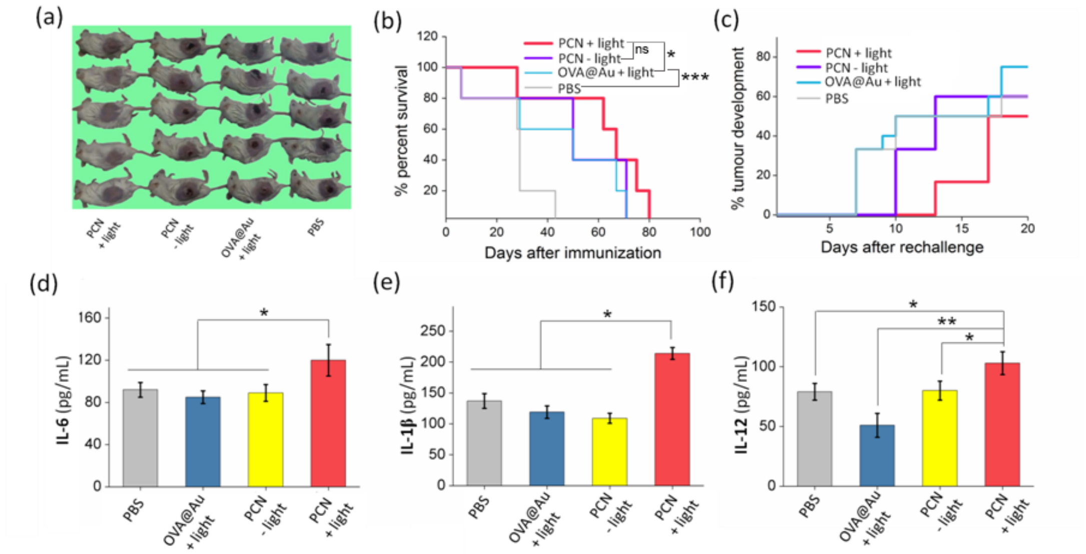

Figure S6. Mild heat improved immunotherapy effect. (a) Photograph of mice at day 15th after treatment. (b) The survival rate of the mice after different treatments. Statistical analysis was performed by the log-rank test, * p<0.05, ** p<0.01, ***p<0.001. (c) A secondary rechallenge with 4T1 tumor cells was administered to mice 7 days after different treatments. (d)-(f) Cytokine levels in serum from mice 7 days after different treatments. The data are presented as means ± s.d. (n=5).; Statistical analysis was performed by one-way factorial ANOVA. * p<0.05, ** p<0.01, ***p<0.001.

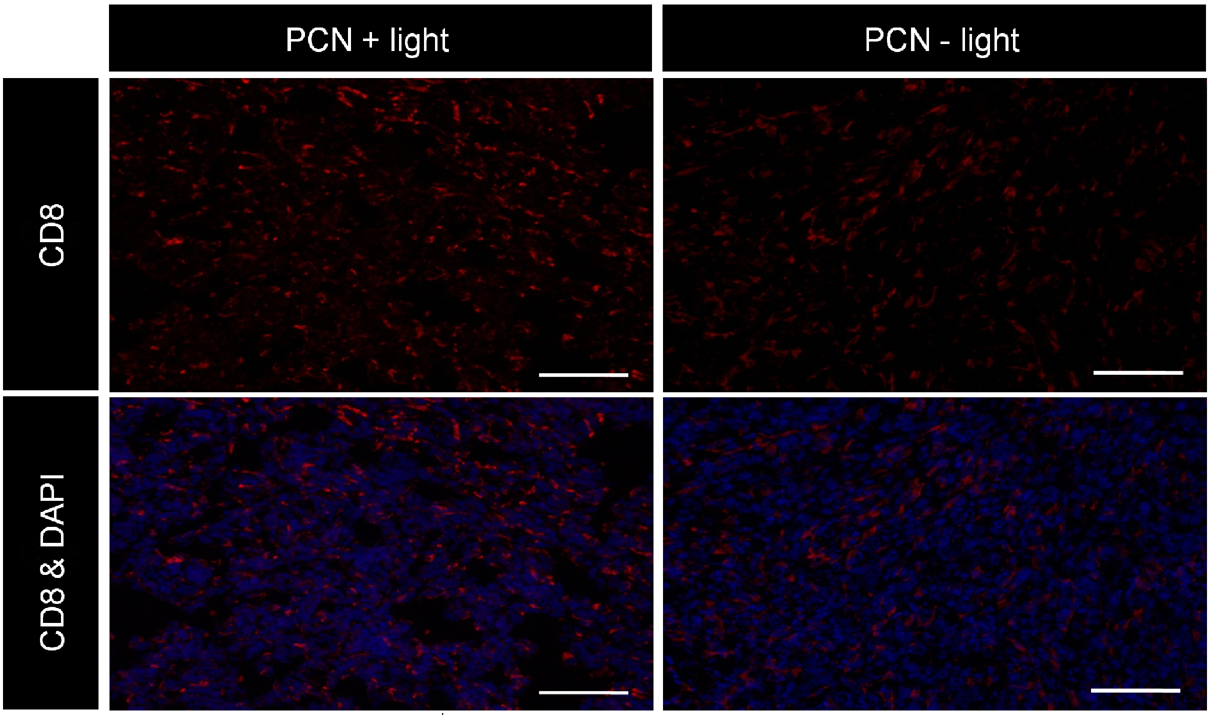

Figure S7. The expression of $CD8^+$ T cells in tumor by immunofluorescence 7 days after intratumoral injection of PCN with and without light. All scale bars are 100 μm.
